# Editorial: Molecular Characterization of Thyroid Lesions in the Era of “Next-Generation” Techniques

**DOI:** 10.3389/fendo.2022.955185

**Published:** 2022-06-29

**Authors:** Claudio Bellevicine, Alessia Ciarrocchi, Alex Friedlaender, Umberto Malapelle, Dario de Biase

**Affiliations:** ^1^ Department of Public Health, University of Naples Federico II, Naples, Italy; ^2^ Laboratory of Translational Research, Azienda Unità Sanitaria Locale-IRCCS di Reggio Emilia, Reggio Emilia, Italy; ^3^ Oncology Department, University Hospital of Geneva, Switzerland; Oncology Service, Clinique Générale Beaulieu, Geneva, Switzerland; ^4^ Department of Pharmacy and Biotechnology (FaBiT), University of Bologna, Bologna, Italy; ^5^ Solid Tumor Molecular Pathology Laboratory, IRCCS Azienda Ospedaliero-Universitaria di Bologna, Bologna, Italy

**Keywords:** thyroid cancer, next generating sequencing, thyroid pathologies, molecular techniques, tumor mutation burden, immune system

Thyroid pathologies are the most frequently diagnosed endocrine disorders, of which thyroid carcinoma is the most common malignant endocrine disease. Moreover, the detection of small thyroid nodules by neck ultrasonography has led to an increased incidence of thyroid cancer. The evidence that mortality for thyroid tumors remains stably low indicates a need for precise parameters to distinguish clinically aggressive thyroid nodules. It is now apparent that thyroid tumors show a very strong correlation between genotype and phenotype, a correlation that is much stronger than that observed in tumors of many other organs.

Although the Bethesda System for Reporting Thyroid Cytopathology has improved the interobserver agreement for the categorization of thyroid fine-needle aspirates, the indeterminate categories such as the atypia of undetermined significance/follicular lesion of undetermined significance (AUS/FLUS) and follicular neoplasm/suspicious for follicular neoplasm (FN/SFN) still pose challenges for both cytopathologists and clinicians. Huang et al. investigated the malignancy risk of 22 thyroid nodules reported as Bethesda category III (AUS/FLUS) on initial Fine-needle aspiration specimen *[Surgical Outcome and Malignant Risk Factors in Patients With Thyroid Nodule Classified as Bethesda Category III]* . They found that a malignant diagnosis in patients with Bethesda category III thyroid nodules was significantly and independently related to microcalcifications, shape, diameter, and nodule goiter. Moreover, thyroid nodules classified as Bethesda category III were significantly associated with preoperative serum TGAb and A-TPO.

The introduction of multigene molecular panels and next-generation sequencing technologies has been thoroughly investigated in the hopes of improving diagnostic accuracy among indeterminate thyroid nodules (1–5). In a cohort of 188 indeterminate thyroid nodules, both TIR3A (56.4%) and TIR3B (43.6%), analyzed using ultrasound (US) and a seven-gene panel test, Capezzone et al. have observed that the US score allows us to discriminate between TIR3A (AUS/FLUS) nodules in which a conservative approach may be used and those in which molecular testing may be indicated. On the contrary, the risk of thyroid cancers in TIR3B (FN/SFN) nodules remains high regardless of US score and mutational status *[The Combination of Sonographic Features and the Seven-Gene Panel May be Useful in the Management of Thyroid Nodules With Indeterminate Cytology]*.

Papillary thyroid cancer (PTC) is the most frequent subtype of thyroid cancer and is characterized by common molecular alterations, such as *BRAF* mutations (6). However, the prognosis of patients with advanced PTCs remains poor, and unveiling molecular mechanisms of neoplastic progression is critical for improving the clinical management of patients with advanced PTCs. Geng et al. have observed that the *SHCBP1* (SHC SH2 Domain-Binding Protein 1) gene was significantly up-regulated in PTC tumor tissues. Patients with high *SHCBP1* expression levels tend to have more lymph node metastases and the mRNA level of *SHCBP1* was negatively associated with patients’ disease-free survival rates. In this study, *in vitro* and *in vivo* assays demonstrated that knock-down of SHCBP1 significantly inhibits PTC cell proliferation, cell cycle, invasion, and migration, suggesting that SHCBP1 might be involved in PTC carcinogenesis and progression *[SHCBP1 Promotes Papillary Thyroid Carcinoma Carcinogenesis and Progression Through Promoting Formation of Integrin and Collagen and Maintaining Cell Stemness]*

In thyroid cancers, *TERT* promoter mutations are associated with lower disease-free survival and a higher frequency of metastases (7, 8). da Costa et al. compared three different strategies for investigating the prevalence of *TERT* promoter mutations in a cohort of PTC *[Advances in Detecting Low Prevalence Somatic TERT Promoter Mutations in Papillary Thyroid Carcinoma]*: Allelic Discrimination Assay, Sanger sequencing, and Droplet Digital PCR (ddPCR).

Sanger sequencing proved to be the less sensitive technique, failing to detect *TERT* promoter mutations in 8% of samples that were positive by an allelic discrimination assay. Intriguingly, ddPCR has a higher sensitivity, detecting *TERT* promoter mutations in 20% of samples that were negative for *TERT* mutations using the other two techniques. Moreover, ddPCR would allow also the absolute quantification of copy number alterations, a distinct mechanism that results in *TERT* upregulation (9). The authors emphasize the use of ddPCR in the clinical practice of thyroid nodules, mainly in the context of aggressive and advanced thyroid carcinomas.

Tumor mutation burden (TMB) is a quantitative measurement of the total number of somatic non-synonymous mutations in the coding area of the tumor genome (10). TMB is predictive of the activity of immune checkpoint inhibitor therapies in several cancers (11). Guo et al. downloaded RNA-seq and DNA-seq datasets of PTC patients from The Cancer Genome Atlas (TCGA) database and determined the differentially expressed genes between the high and low TMB thyroid groups *[Tumor Mutation Burden Predicts Relapse in Papillary Thyroid Carcinoma With Changes in Genes and Immune Microenvironment]*. A higher TMB score was related to a worse prognosis and showed a higher level of oxidative phosphorylation genes. With regards to the immune microenvironment, in the high TMB group, the CD8^+^ T cells and M1 macrophages were significantly lower than that in the low TMB group. These data support the evidence that TMB is an independent prognostic factor in PTC and suggest that TMB may be clinically meaningful for monitoring the recurrence to shorten the follow-up time of PTC (Guo et al.).

The immune system plays a key role in cancer initiation and progression and neoplastic cells may acquire the capacity to evade the immune response. In the review by Menicaliet al., there is a comprehensive and updated assessment of the current immune landscape in thyroid tumors *[Immune Landscape of Thyroid Cancers: New Insights]*. The thyroid tumor microenvironment is different according to thyroid tumor histotypes. PTC immune infiltrates are characterized by Tumor-Associated Macrophages, Dendritic Cells, a high density of Tumor-Associated Mast Cells, Tumor-Associated Neutrophils, Natural Killer Cells, and CD8+ and CD4+ T cells (Menicaliet al.). Anaplastic thyroid carcinomas’ immune infiltrate is composed of a high density of Tumor-Associated Macrophages, Tumor-Associated Mast Cells, Myeloid-Derived Suppressor Cells, Natural Killer Cells, and CD8+ and CD4+ T cells. Few data are available about immune cells in poorly differentiated Thyroid carcinomas, but the latter are known to display Tumor-Associated Macrophages and Tumor-Associated Mast Cells (Menicaliet al.). As the understanding of the immune microenvironment in thyroid cancer improves, it will contribute to the development of personalized therapeutic solutions to counteract failure and/or resistance to immunotherapy.

This Research Topic has highlighted that, even if our understanding of the molecular pathology of thyroid cancer has progressed significantly, improving care, there is still much room for improvement. Ongoing research will hopefully improve diagnostic accuracy and lead to novel therapeutic approaches, as advanced diagnostic strategies better classify thyroid tumors and predict their biological behavior, prognosis, or response to therapy.

## Author Contributions

All authors listed have made a substantial, direct, and intellectual contribution to the work and approved it for publication.

## Conflict of Interest

The authors declare that the research was conducted in the absence of any commercial or financial relationships that could be construed as a potential conflict of interest.

## Publisher’s Note

All claims expressed in this article are solely those of the authors and do not necessarily represent those of their affiliated organizations, or those of the publisher, the editors and the reviewers. Any product that may be evaluated in this article, or claim that may be made by its manufacturer, is not guaranteed or endorsed by the publisher.
